# A robust, low- to medium-throughput *prnp *genotyping system in sheep

**DOI:** 10.1186/1471-2334-4-30

**Published:** 2004-09-02

**Authors:** Johannes Buitkamp, Jördis Semmer

**Affiliations:** 1 Bavarian State Research Center for Agriculture, Institute of Animal Breeding, Prof.-Dürrwaechter-Platz 1, 85586 Poing, Germany

## Abstract

**Background:**

In many countries breeding programs for resistance to scrapie in sheep are established. Therefore, the demand on genotyping capacities of the polymorphisms of the prion protein gene (*prnp*) relevant to presently known disease associations and EU regulations is steadily increasing. Most published typing methods are not well suited for routine typing of large sample numbers in smaller service laboratories for different reasons: they require partly manual data processing, sophisticated and sensitive protocols, high efforts regarding time and manpower, multiple step reactions or substantial hardware investments. To overcome these drawbacks, we developed a *prnp *typing method that is based on a `multiplex amplification refractory mutation system' (ARMS) reaction.

**Methods:**

In this study we combined the amplification refractory mutation system (ARMS) with standard fluorescent based fragment length analyses method to develop a *prnp *genotyping method (PRNP ARMS).

**Results:**

By optimised primer design it was possible to type the 4 relevant single nucleotide polymorphisms (SNPs) in the *prnp *simultaneously in one multiplex reaction. Automated fragment length analysis enabled automated allele designation. Suitability of the PRNP ARMS for routine application was proven by typing samples with known genotypes and larger sample numbers from half-sib families.

**Conclusion:**

The ARMS PRNP typing method established in this study is universally suited for a broad range of typing projects with different requirements. It provides an efficient and inexpensive diagnostic mutation analysis that will improve the quality of *prnp *genotyping compared with other low-cost methods. It can be implemented by most molecular genetic laboratories using standard equipment.

## Background

Scrapie is a contagious prion disease [1] of sheep and goat. In contrast to BSE, there is no evidence for a transmission to humans. Nevertheless, BSE is experimentally transmittable to sheep and the resulting disease can not be distinguished from scrapie [2,3]. Even though BSE has not been found in farmed sheep yet, the possibility that BSE occurs in "scrapie" diseased sheep can not be excluded. Therefore, scrapie control programs were implemented in many countries. Since no vaccine or therapeutic means is currently available, these programs rely on selective breeding for scrapie resistance. Susceptibility to scrapie is largely controlled by three polymorphic amino acid positions (136, 154, 171) of the ovine prion protein gene (*prnp*) [4] and reliable genotyping of the corresponding DNA polymorphism is required as a basis for selection decisions.

For determination of *prnp *alleles or haplotypes, there are several typing techniques applied today. Direct sequencing covering the region of exon 2 encoding amino acid positions 136, 154 and 171 is the most accurate method, that enables the typing of additional and detection of hitherto unknown polymorphisms. Other methods are classical PCR-restriction fragment length polymorphism (RFLP)-typing [5,6], DNA strand conformation polymorphism detection, denaturing gradient gel electrophoresis (DGGE) [7], PCR-single-strand conformational polymorphism (PCR-SSCP) [8], hybridisation with allele-specific oligonucleotides [9,10], primer extension [11], and so on. For high-throughput genotyping matrix assisted laser desporption/ionisation – time of flight (MALDI-TOF) systems (e.g. for bovine *prnp *[12] and the LGC-web page [13]), taqman^® ^(Applied Biosystems, CA) [14], and pyrosequencing (Biotage AB, Uppsala, Sweden) are used, but methodologically details of these methods are not very well documented in the literature.

The amplification refractory mutation system (ARMS) method is well established [15] and has been applied not only to single nucleotide polymorphism (SNP) detection but also to haplotype determination [16]. In the present work we combined the use of fluorescence labelled oligonucleotides with ARMS technology for the simultaneous detection of 4 SNPs without using common primers. Based on this PRNP ARMS method we developed a cost-effective, well-reliable and low- to high-throughput technology for *prnp *typing that can easily be adopted by a wide range of other laboratories.

## Methods

### DNA-Samples

EDTA-blood or Typifix^®^-tissue samples were collected from half-sib families from three common sheep breeds in Bavaria. All lambs were born in the years 2001 and 2002. DNA was isolated by using the E.Z.N.A. Blood DNA Mini Kit (#12-3482-03, PEQLAB Biotechnologie GmbH, Erlangen, Germany) or the NucleoSpin^® ^Multi-96 Tissue kit (MACHEREY-NAGEL GmbH & Co. KG, Düren, Germany), respectively.

### PRNP-ARMS

#### Design of Primers

**Figure 1:**
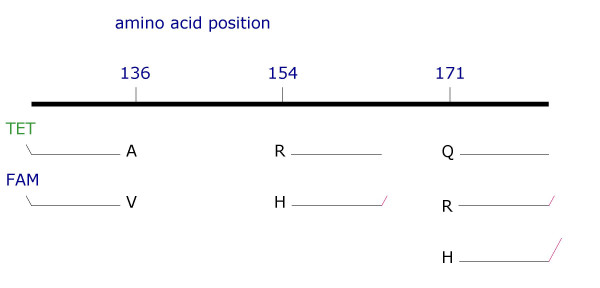
**Scheme of PRNP ARMS-PCR design. **PrP amino acid positions are given above. The allele-specific up-primers (discrimination of A-V at codon 136) are fluorescence labelled, whereas down primers (discrimination of R-H and Q-R-H at codon 154 and 171, respectively) are of different lengths (tails are shown as red lines).

An overview of primer locations is given in Fig. 1. Initially, allele-specific primers were designed that differed only at the 3'-nucleotide (given in bold letters in Table 1). Using these primers it was not possible to obtain reliable discrimination of alleles. Especially the simultaneous use of primers for alleles Q-171 and R-171 resulted in cross-amplification products. Therefore, a number of additional primers were tested to optimise allele discrimination. Destabilizing mismatches were introduced at the first or second penultimate base in addition to the allele-specific base at the 3' termini of the primers (ARMS principle, indicated by small letters in Table 1). The sense primers specific for the alleles at amino acid position 136 were labelled with two different fluorochromes, 4, 7, 2', 7'-tetrabetweenchloro-6-carboxyfluorescein (TET) and 6-carboxyfluorescein (FAM) (Table 1, Fig. 1). That allows the detection and discrimination of the alleles by standard gel electrophoresis on a fluorescent sequencer. Pieces of neutral sequence [17] were added to the 5'-end (underlined in Table 1) of the unlabeled antisense primers for amino acid positions 154 and 171. These enable the differentiation of the alleles by different lengths. Mismatches between primers were deliberately introduced at the 5' region in these neutral sequences to avoid jumping amplification products. For a delineation of primer sequences with a reference sequence (Fig. 3).

#### PCR-reaction

For the ARMS reaction haplotypes were amplified from 2 μl of DNA solution with standard buffer conditions, 1.5 mM MgCl_2_, dNTP's (25 nM each), and 0.75 units of HotStar-taq polymerase (Qiagen, Hilden, Germany) in a final volume of 10 μl on a t-gradient 96-well thermocycler (Biometra, Göttingen, Germany). Primer concentrations were 10 nmol each. Cycling was for 15 min at 95°C, [0.5 min at 94°C, 1 min at 62°C, 1 min at 74°C]33× without final extension.

#### Fragment analysis

Fragment lengths of ARMS amplification products were analysed on an ABI PRISM^® ^310 genetic analyser (Applied Biosystems, Foster City, CA) with 5 sec injection time, 15 kV, 60° and 18 min runtime using 36 cm capillaries. Allele designations were generated automatically using the Genotyper^® ^software (V. 2.5, Applied Biosystems) as described [18]. The data were imported into an Microsoft Access database. Standard DNA samples representing different alleles were included in the assay to control each PCR mix and reaction. As an additional control a sql-query was used to flag all genotypes transferred to the database that were not compatible with the 15 known standard genotypes (e.g. peaks from FAM in the range of 153 bp) for retyping.

### Comparative sequencing

Part of *prnp *exon 3 coding for amino acids 47 to 246 of the prion protein was amplified in 10 μl reaction volume using 2 μl of genomic DNA, 0.05 μM of each primer (5'-tcc tgg agg caa ccg cta tc-3' and 5'-gga gga tca cag gag ggg aag-3'), 50 μM of each dNTP, 0.5 units of HotStar-taq polymerase (Qiagen, Hilden, Germany), and reaction buffer containing 1.5 mM MgCl_2_. Cycling-conditions on a Biometra T gradient 96-well thermocycler were: 15 min at 95°C, [0.5 min at 95°C, 1 min at 60°C, 0,5 min at 72°C]35× and 10 min, 60°C final extension. Before direct sequencing PCR-reactions were purified using the 96-well MultiScreen-PCR plates (Millipore, Bedford, MA). Sequencing was performed using BigDye V 2.0 terminator cycle sequencing kit (Applied Biosystems, Foster City, CA). Sequencing analysis was run on an ABI PRISM^® ^310 genetic analyser.

## Results

### Development of the PRNP ARMS method

**Figure 2:**
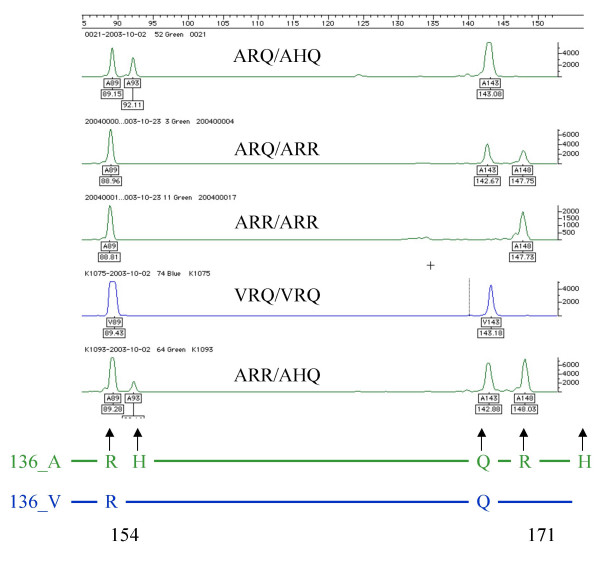
**Electropherograms from 5 *PRNP *genotypes. **The "green" lanes correspond to TET labelled fragments, the "blue" lines to FAM labelled products. The *PRNP *genotype of each individual is given above each lane. Only the informative lanes (no peaks were observed in the "blue" lane of A136 and no peaks were observed in the "green" lane of V136 homozygotes, respectively) are shown. The scheme at the bottom shows how the peaks relate to the alleles.

**Figure 3:**
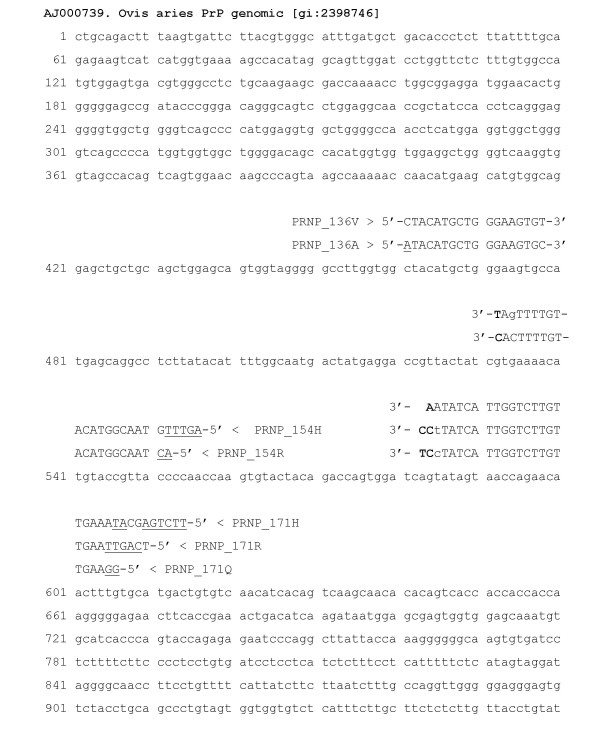
**Location of ARMS Primers within the reference sequence. **Primer sequences are given above the sequence representing the "wild-type" allele (Genbank accession number AJ000739). The 5 prime and 3 prime ends are indicated. Small letters indicate deliberately introduced mismatches; underlining indicate pieces of neutral sequence that were deliberately added to elongate the primer.

We have established a multiplex `amplification refractory mutation system' (ARMS) based method for the identification of *prnp *136-154-171 genotypes in sheep. The PRNP ARMS method was developed using standard DNAs representing all five main haplotypes. In a first stage, different primer pairs were tested for allele specific amplification using an anneal-temperature gradient. By redesign of primers for amino-acid positions 154 and 171 and introducing deliberate mismatches close to the 3' end an optimised primer set was established (Table 1) allowing multiplex, allele-specific amplification. "False positive fragments" (peaks above the background signal generated by primers complementary to alleles that were not present in the corresponding sample) were not observed using the optimised primer set. Nevertheless, the average peaks heights differed between alleles. For example the average peak heights of the allele Q-154 was 1.5 times higher than that of R-154. The ratio of peak heights in heterozygous individuals differed from 0.5 to 2.5 fold (compare Fig. 2). These were observed when different PCR assays with low number of samples and small volumes of master-mix are compared. The peak height differences depend mainly on the relative primer concentrations and can be avoided e.g. by using premixed primer solutions containing all ARMS primers. For automated "base calling" the minimum peak height was set to 100 (in practice most samples gave peak heights well above 1000).

### Proof-of-principle

To provide a proof-of-principle the same 140 sheep DNAs were analysed by direct PCR sequencing and by PRNP ARMS. Typing results were identical with both methods. In a second step, 420 sheep from half-sib families and 20 samples from the international sheep and goat DNA typing comparison test 2003 of the International Society of Animal Genetics were analysed by the PRNP ARMS method. All genotypes followed the rules of Mendelian inheritance and no deviation from the main 5 haplotype patterns was observed. Allele frequencies derived from these animals from three different Bavarian breeds are shown in Table 2. Paternity of all lambs was checked with a set of 9 microsatellites (data not shown).

Typical electropherograms representing different haplotype combinations are shown in Fig. 2. Allele length determination was highly reproducible (Table 3). The range of individual peaks from each individual allele was well below +/-1 bp allowing unambiguous allele-calling and automatic generation of results tables using the genotyper software. Furthermore, the PRNP ARMS reaction worked fairly stable with different DNA qualities. The quality and concentration of the DNAs that were isolated from tissue varied widely (5 – 50 ng/μl, various degrees of fragmentation). Failed PCR reactions (e.g. one primer missing) were identified by analysing the standard samples. Failed individual samples (e.g. due to low DNA concentrations or failure of the fragment analyses run) were easily identified by missing or very low and out-of-range peaks. Nevertheless, the proportion of samples that had to be retyped was well below 1%.

## Discussion

The PRNP ARMS genotyping method has several advantages. It is based on a one step reaction using competitive allele discrimination. The fragments can be analysed on an automated sequencer that allows data to be directly transferred to a database. The reaction proved to be highly specific and no false negative or positive results were observed yet. Furthermore, it is robust with respect to variations of DNA quality and, since no further purification or reaction is necessary, the costs for consumables are low. The ARMS method allows the determination of partial (136-154 and 136-171, compare Fig. 1) *prnp *haplotypes, thereby facilitating the detection of 'complex' prnp genotypes that do not match one of the 15 standard genotype patterns. If necessary, it can easily be extended to complete haplotype determination by adding additional labelled forward primers for position 154 (for haplotype 154-171).

All methods currently available for SNP typing are inherent sensitive to nucleotide changes within the primer attachment or restriction sites. The resulting mismatches can cause inconclusive typing results or null alleles, as frequently observed with microsatellite loci. Therefore, it is crucial to be aware of this phenomenon and to take measures to reduce the risk of mistyping. The primers for PRNP ARMS were carefully checked against a *prnp *in-house database containing 16 published and 11 unpublished (mainly from rare German and Spanish breeds) alleles. No known polymorphism interfere with the attachment of the selected ARMS primers. Nevertheless, the recently described rare polymorphism K-171 [19] with unknown effect to scrapie resistance is not included in the standard PRNP ARMS set (that gives Q-171 as typing result for this allele) and requires one additional specific oligonucleotide. This oligonucleotide should be added if the respective breeds (mainly hair breeds [19]) are genotyped. When further polymorphisms associated with resistance to scrapie will be identified appropriate oligonucleotides can easily be added. Unlabelled additional oligonucleotides can be used when SNPs upstream of the amino acid position 136 shall be typed. Nevertheless, the inclusion of positions downstream of position 136 would require additional labelled oligonucleotides. A third fluorescent dye might be useful especially when the lengths of the fragments interfere with on of the other alleles.

The PRNP ARMS method is highly flexible with respect to scale and instrumentation. It is easily adoptable from low- to medium-throughput typing system using identical reaction conditions. In principle, all primer combinations can even be performed as single reactions that can be analysed on agarose gels (data not shown). Nevertheless, the reliability of genotyping results improves when competitive reaction conditions and commonly available DNA sequencing machines are used. Since all reactions can be performed in microtiter plates, the ARMS reaction can easily be adopted for a pipetting robot. Together with the one step reaction and the automated data transfer, the risk of cross-contamination or interchanging of samples is minimized. The runtime of 18 min results in 25 min analyses time per sample on the ABI 310 and could be optimised by using shorter capillaries. When using the standard run conditions on the smallest, single capillary sequencer the throughput is limited to about 55 samples per working day but can be scaled up to more than 5000 samples per day by using a 96-well capillary sequencer. That should be sufficient even for large, existing breeding programs. PRNP ARMS can be multiplexed with other typing systems (microsatellite or SNP), since it is analysed using standard fragment analysis technique. Moreover, it is suitable for further applications as e.g. allele frequency estimation from pooled DNA [20] for investigating rare breeds.

## Conclusions

An easy and robust one-step *prnp *typing method has been established that is universally suited for a broad range of typing projects with different requirements. This typing method was developed by optimising ARMS primers and combining these with standard fragment length analyses. The method provides an efficient and inexpensive diagnostic mutation analysis that can be implemented by most molecular genetic laboratories using standard equipment. It should contribute to reliable and economic genotyping of the ovine *prnp *by the many smaller labs throughout Europe.

## Competing interests

The authors declare that they have no competing interests.

## Authors' contributions

J.B. designed the project and protocols involved, performed analysis of results and drafted this manuscript. J.S. conceived the ARMS reaction, fragment analysis and DNA sequencing.

## Pre-publication history

The pre-publication history for this paper can be accessed here:



## Figures and Tables

**Table 1:** ARMS primers used to type the ovine PRNP

Name	Sequence of Primer 5'-3'	Allele	Length/Label	Fragment Length
PRNP_136A	ATACATGCTGGGAAGTG**C**	A^136^	18 bp/TET	-
PRNP_136V	CTACATGCTGGGAAGTG**T**	V^136^	18 bp/FAM	-
PRNP_154R	ACTAACGGTACATGTTTTCA**C**	R^154^	21 bp/-	92 bp
PRNP_154H	AGTTTGTAACGGTACATGTTTTgA**T**	H^154^	25 bp/-	96 bp
PRNP_171Q	GGAAGTTGTTCTGGTTACTATc**CT**	Q^171^	24 bp/-	146 bp
PRNP_171R	TCAGTTAAGTTGTTCTGGTTACTATt**CC**	R^171^	28 bp/-	150 bp
PRNP_171H	TTCTGAGCATAAAGTTGTTCTGGTTACTATA**A**	H^171^	32 bp/-	154 bp

Small letters indicate deliberately introduced mismatches (ARMS principle); underlining indicate pieces of neutral sequence that were added to elongate the primer to allow allele-discrimination by capillary electrophoresis.

**Table 2:** *PRNP *allele frequencies (%) as observed in three sheep breeds common in Bavaria

		breed	
Allele	ML	SK	SU
AHQ	7.68	0.00	1.96
ARH	0.18	0.00	0.00
ARQ	82.14	24.58	27.45
ARR	9.82	69.49	66.67
VRQ	0.18	5.93	3.92

ML, Merinolandschaf; SK, German Blackheaded Mutton; SU, Suffolk.

**Table 3:** Statistics for peak lengths of the PRNP ARMS system

			Calculated peak sizes^#^			
Dye	Oligonucleotide	Fragment length	Mean	StdDev	Min	Max
TET	PRNP_154R	92 bp	89.16	0.12	88.91	89.70
	PRNP_154H	96 bp	92.13	0.09	91.98	92.45
	PRNP_171Q	146 bp	142.91	0.12	142.67	143.48
	PRNP_171R	150 bp	148.05	0.18	146.72	148.42
	PRNP_171H	154 bp	153.13	0.72	152.31	153.74
FAM	PRNP_154R	92 bp	89.56	0.13	89.36	89.84
	PRNP_154H	96 bp	np	np	np	np
	PRNP_171Q	146 bp	143.29	0.11	143.13	143.48
	PRNP_171R	150 bp	np	np	np	np
	PRNP_171H	154 bp	np	np	np	np

^#^peak lengths as calculated using the genotyper software from all genotype results in the database; np: not present in the dataset
